# Prevalence and mechanisms of antibiotic resistance in *Escherichia coli* isolated from mastitic dairy cattle in Canada

**DOI:** 10.1186/s12866-021-02280-5

**Published:** 2021-07-31

**Authors:** Satwik Majumder, Dongyun Jung, Jennifer Ronholm, Saji George

**Affiliations:** 1 Department of Food Science and Agricultural Chemistry, McGill University, Macdonald Campus, 21111 Lakeshore Ste Anne de Bellevue, H9X 3V9 Quebec, Canada; 2 Department of Animal Science, McGill University, Macdonald Campus, 21111 Lakeshore Ste Anne de Bellevue, H9X 3V9 Quebec, Canada

**Keywords:** Antimicrobial resistance (AMR), *E. coli*, Bovine mastitis, Antibiotics, Heavy-metals, Efflux pump, ß-lactamase enzyme, Biofilm, Whole-genome sequencing

## Abstract

**Background:**

Bovine mastitis is the most common infectious disease in dairy cattle with major economic implications for the dairy industry worldwide. Continuous monitoring for the emergence of antimicrobial resistance (AMR) among bacterial isolates from dairy farms is vital not only for animal husbandry but also for public health.

**Methods:**

In this study, the prevalence of AMR in 113 *Escherichia coli* isolates from cases of bovine clinical mastitis in Canada was investigated. Kirby-Bauer disk diffusion test with 18 antibiotics and microdilution method with 3 heavy metals (copper, zinc, and silver) was performed to determine the antibiotic and heavy-metal susceptibility. Resistant strains were assessed for efflux and ß-lactamase activities besides assessing biofilm formation and hemolysis. Whole-genome sequences for each of the isolates were examined to detect the presence of genes corresponding to the observed AMR and virulence factors.

**Results:**

Phenotypic analysis revealed that 32 isolates were resistant to one or more antibiotics and 107 showed resistance against at least one heavy metal. Quinolones and silver were the most efficient against the tested isolates. Among the AMR isolates, AcrAB-TolC efflux activity and ß-lactamase enzyme activities were detected in 13 and 14 isolates, respectively. All isolates produced biofilm but with different capacities, and 33 isolates showed α-hemolysin activity. A positive correlation (Pearson r = + 0.89) between efflux pump activity and quantity of biofilm was observed. Genes associated with aggregation, adhesion, cyclic di-GMP, quorum sensing were detected in the AMR isolates corroborating phenotype observations.

**Conclusions:**

This investigation showed the prevalence of AMR in *E. coli* isolates from bovine clinical mastitis. The results also suggest the inadequacy of antimicrobials with a single mode of action to curtail AMR bacteria with multiple mechanisms of resistance and virulence factors. Therefore, it calls for combinatorial therapy for the effective management of AMR infections in dairy farms and combats its potential transmission to the food supply chain through the milk and dairy products.

**Supplementary Information:**

The online version contains supplementary material available at 10.1186/s12866-021-02280-5.

## Background

Bovine mastitis is a common and very costly infectious disease that has a high prevalence in the global dairy industry. In the US and Canada, bovine mastitis results in a net annual loss of about $2 billion (USD) and $794 million (CAD), respectively [1]. Clinical management of mastitis is challenging because of the multiple etiological agents including *Staphylococcus aureus*, non-aureus staphylococci (NAS), *Escherichia coli*, *Klebsiella spp.*, and *Streptococcus spp.* [2]. *E. coli* is one of the most common environmental bovine mastitis pathogens, found in almost 80 % of the cases of coliform mastitis which infects the mammary glands during the dry period [3]. While intramammary infection (IMI) involving *E. coli* are usually short-lived, 5–20 % are reported to persist due to their ability to adhere and survive intracellularly [4, 5].

Antibiotics have been used extensively in animal agriculture for infection control and as growth promoters [6]. Heavy metals are also widely used in farms as therapeutics, in feed, and to improve reproductive efficiency [7]. Indiscriminate use of antimicrobials in farms has been suspected as a major factor in the emergence of antimicrobial resistance (AMR) among pathogenic bacteria. Prevalence of AMR bacteria in IMI is not only a challenge for clinical management of mastitis but also a public health concern is given the possibilities of transfer of AMR bacteria or genetic determinants from animals to humans *via* the food chain [7–9].

Identified mechanisms of resistance to clinically important drugs used in bovine mastitis treatment in Canada include extended-spectrum β-lactamases (ESBLs), plasmid-mediated AmpC β-lactamases, carbapenemases, and generalized efflux pump activity [10, 11]. Due to a wide range of substrate specificity and high levels of constitutional expression under physiological conditions, the RND-based tripartite efflux pump- AcrAB-TolC is considered the most significant contributor to intrinsic multidrug resistance in *E. coli* [11]. In addition to AMR, other virulence factors favor the survival of bacteria in host tissue. For instance, *E. coli* survives and colonizes bovine udder by hemolysis and biofilm formation [5].

Biofilms protect resident bacteria from the antibiotic activity and host defenses leading to bacterial persistence in hostile host tissues and increase the risk of disease transmission [2, 3]. Secretory virulence factors, such as hemolysin, are also reported to be responsible for pore formation and cellular necrosis which involve a cell-to-cell interaction during bacterial biofilm formation, increase in inflammatory responses, and decrease in macrophage function [3, 5].

The Canadian Bovine Mastitis Research Network maintains a culture collection of bacterial isolates from mastitis infected dairy cows - Mastitis Pathogen Culture Collection (MPCC). These isolates were collected from 91 dairy farms across Canada over 2 years in 2007 and 2008 [12]. In this study, we assessed the prevalence of AMR and virulence characteristics of 113 *E. coli* isolates obtained from MPCC using phenotypic assays. Further, the presence of genes corresponding to the identified AMR and virulence characteristics were verified from the whole genome data reported recently [13, 14]. Knowledge about the prevalence of AMR and virulence factors involved in the survival and persistence of *E. coli* causing IMI is pivotal for clinical management of disease as well as for designing new therapeutic agents.

## Results

### Antibiotic and metal resistance profiles of the *E. coli* isolates

**Fig. 1:**
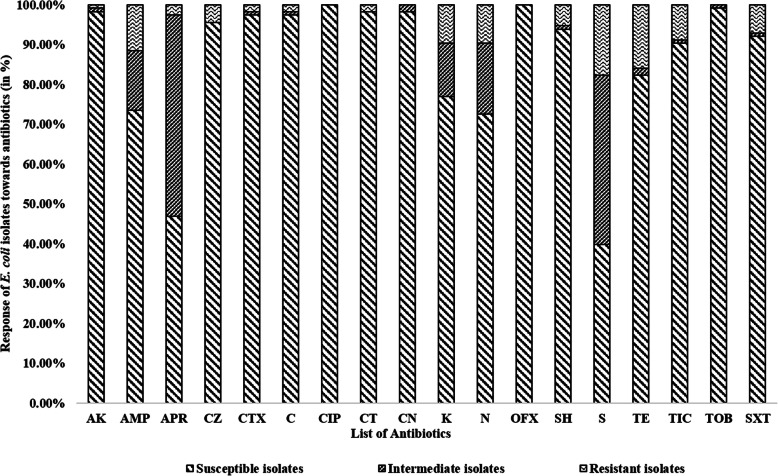
**Response pattern of 113** ***E. coli*** **isolates toward 18 antibiotics**. *E. coli* isolates were subjected to Kirby-Bauer disk diffusion susceptibility tests. The scores, based on CLSI guidelines for susceptibility or resistance to an antibiotic, were generated for each isolate. Abbreviations used - *AK* Amikacin, *AMP* Ampicillin, *APR* Apramycin, *CZ* Cefazolin, *CTX* Cefotaxime, *C* Chloramphenicol, *CIP* Ciprofloxacin, *CT* Colistin, *CN*Gentamycin, *K* Kanamycin, *N* Neomycin, *OFX* Ofloxacin, *SH* Spectinomycin, *S* Streptomycin, *TE* Tetracycline, *TIC* Ticarcillin, *TOB* Tobramycin, *SXT* Trimethoprim/Sulfamethoxazole

Out of 113 isolates, 32 isolates (28.31 %) showed resistance to either single (13/32) or multiple (19/32) antibiotics (Fig. 1). Based on their responses against the antibiotic classes, 13 out of the 32 antibiotic-resistant isolates were labeled as multi-drug resistant isolates, 6 were marked as extensively drug-resistant, whereas the rest 13 isolates were designated to be single drug-resistant (Table 1). The frequency of resistance among the tested *E. coli* isolates was highest towards streptomycin (17.7 %) followed by tetracycline (15.93 %) and ampicillin (11.5 %), whereas less than 10 % resistance was seen towards the remaining antibiotics (supplementary table S2.b.). Out of 113 isolates, 1.76 and 4.42 % of them showed resistance towards cefotaxime and cefazolin, respectively. 1.76 % of the isolates showed resistance against colistin. None of the isolates showed resistance to quinolones (ciprofloxacin and ofloxacin) and aminoglycosides (gentamycin and tobramycin). Out of the 32 resistant isolates, 28.12 and 50.00 % of them were collected from the cattle with mastitis severity score 2 (abnormal milk, swollen quarter) and 3 (abnormal, milk, swollen quarter, and sick cow), respectively.

**Table 1:**
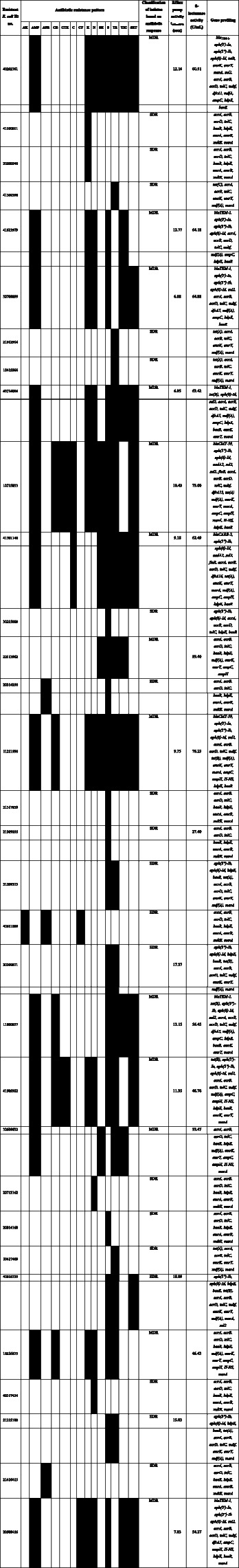
Antibiotic resistance patterns (denoted in Black), efflux pump, ß-lactamase activity, and gene profile of the 32 antibiotic-resistant *E. coli* isolates

Abbreviations used- *AK* Amikacin, *AMP* Ampicillin, *APR* Apramycin, *CZ* Cefazolin, *CTX* Cefotaxime, *C* Chloramphenicol, *CT* Colistin, *K* Kanamycin, *N* Neomycin, *SH* Spectinomycin, *S* Streptomycin, *TE* Tetracycline, *TIC* Ticarcillin, *SXT* Trimethoprim/Sulfamethoxazole, *MDR* Multi-drug resistant, *EDR* Extensively drug-resistant, *SDR* Single drug-resistant

**Fig. 2:**
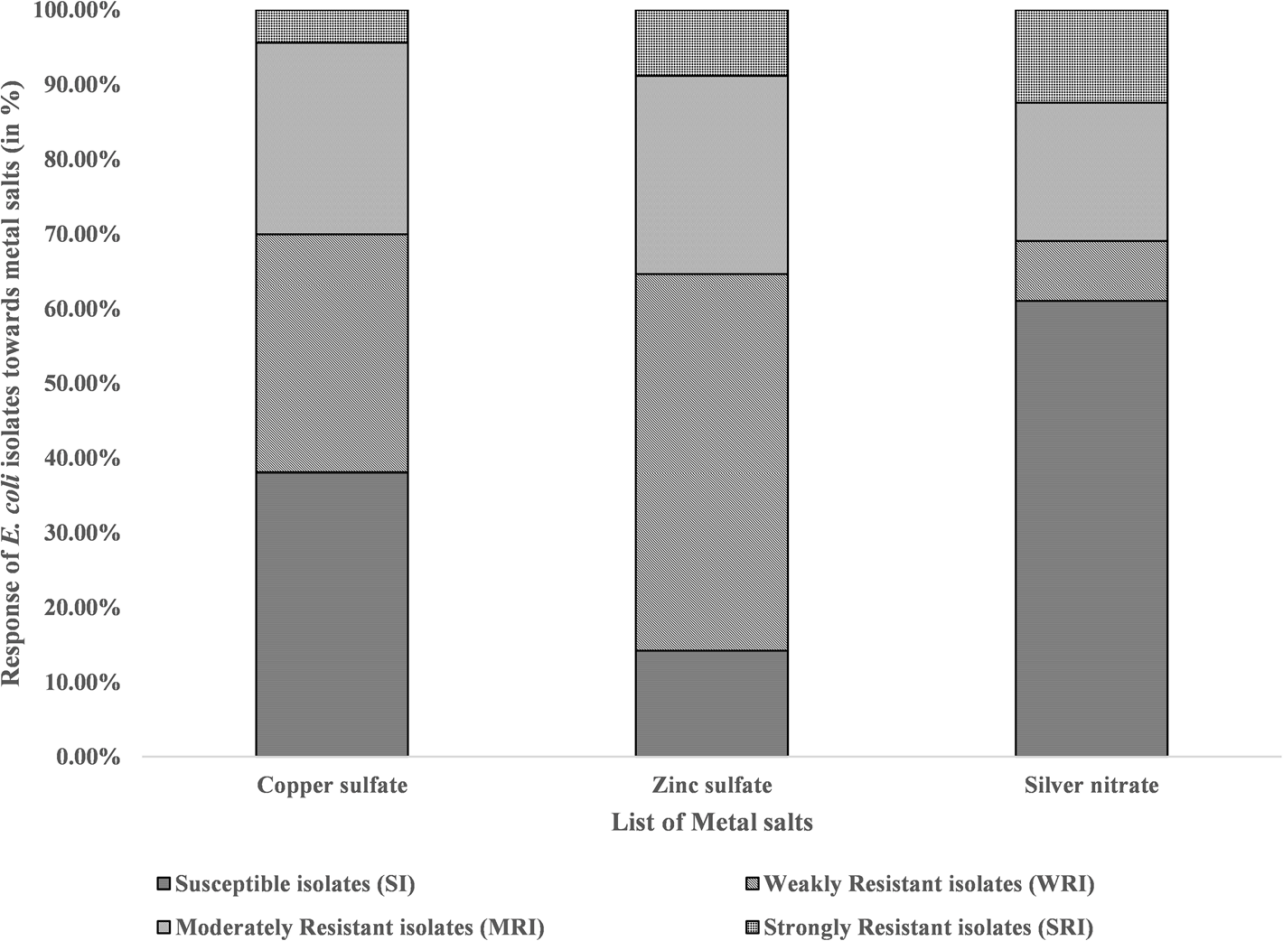
**Response pattern of 113** ***E. coli*** **isolates toward metal salts.** Serial dilutions of metal salts added to MH broth were prepared in 96 well plates and each well was innoculated with live bacteria. After overnight incubation, bacterial viability was assessed using the Resazurin assay. *E. coli *ATCC 25922 was used as a QC strain. The IC50 values of each metal salt against every *E. coli* isolate was calculated using GraphPad Prism 7 software. The IC50 value of each metal salt against the QC strain was considered as the cut-off concentration. E. coli isolates with IC50 values less or equal or non-significant (*p*>0.05) to the cut-off were considered as susceptible whereas, statistically significant (*p*≤0.05) non-susceptible isolates were categorized into weakly resistant isolate(WRI) (QCIC50cut-off < WRI ≤ 1.5 folds of QCIC50cut-off ), moderately resistant isolate (MRI) (1.5 folds of QCIC50cut-off < MRI ≤ 2 folds of QCIC50cut-off) and strongly resistant isolate (SRI) (SRI > 2 folds of QCIC50cut-off)

Of the 113 isolates, 19 isolates were resistant to all the tested heavy metals, 67 isolates showed resistance towards two heavy metals, whereas 21 isolates showed single metal resistance. These bacterial isolates showed the highest resistance towards ZnSO_4_ (85.87 %) followed by CuSO_4_ (61.96 %) and AgNO_3_ (38.93 %) (Fig. 2). In the case of ZnSO_4_, 50.44 % of the isolates showed weak resistance, whereas 26.55 % of the isolates were moderately resistant. Similarly, 31.86 % of the isolates showed weak resistance towards CuSO_4_, whereas 25.66 % were moderately resistant. The least resistance was seen towards AgNO_3_ where 7.96 % of the isolates were weakly resistant, 18.58 % were moderately resistant and the rest showed strong resistance (supplementary table S5.b). Out of 32 antibiotic-resistant isolates, 29 isolates were observed to be resistant towards AgNO_3_ where 40.62 % of them were moderately resistant, 34.37 % showed strong resistance and the rest were weakly resistant. It was followed by ZnSO_4_ (87.50 %) where 50 % of the isolates showed weak resistance, 37.5 % showed moderate resistance and the rest were susceptible. Lastly, 53.13 % of antibiotic-resistant isolates were weakly resistant to CuSO_4_, 37.50 % were susceptible and less than 7 % were either strong or moderately resistant (Table 2).

**Table 2:**
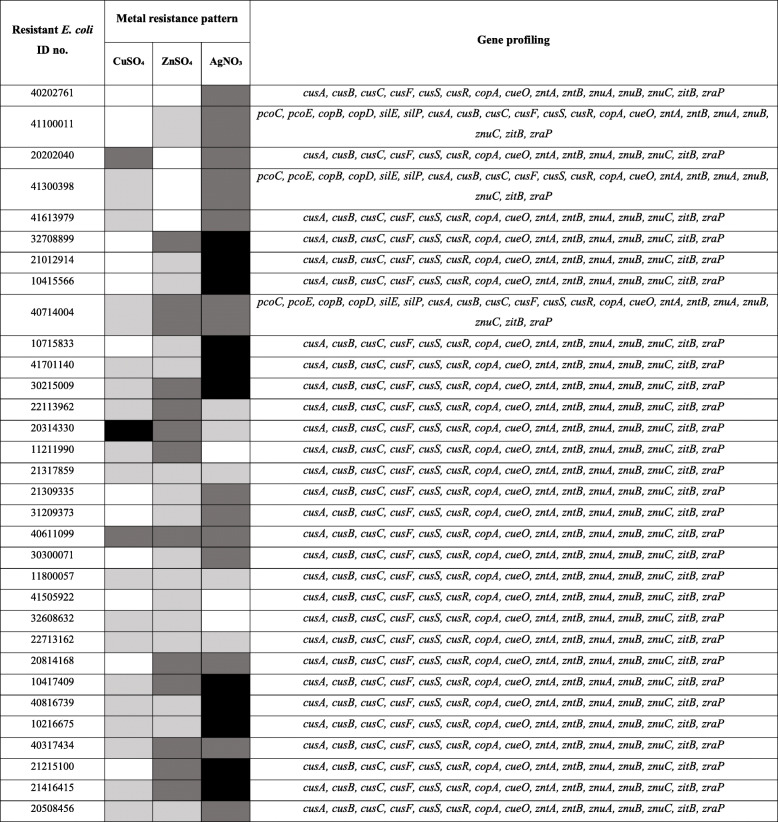
Metal resistance pattern and gene profile of the 32 antibiotic-resistant *E. coli* isolates

Abbreviations used- *CuSO*_*4*_ Copper sulfate, *ZnSO*_*4*_ Zinc sulfate, *AgNO*_*3*_ Silver nitrate. Color codes: Weakly resistant isolates-Light Grey; Moderately resistant isolates-Dark Grey; Strongly resistant isolates-Black

Antibiotic and metal resistance genes were identified from whole genomes of *E. coli* isolates (Tables 1 and 2, supplementary table S4 and S5.b). Clinically important AMR genes were identified from these isolates. For example, ESBL producing genes (*bla*_TEM−B_; 6/113 *bla*_CARB−3_; 1/113), plasmid-mediated AmpC ß-lactamase gene (*bla*_CMY−59_; 2/113), aminoglycoside resistance genes (*aph(3’)-Ia*; 5/113, *aph(3’’)-Ib*; 14/113, *aph(6)-Id*; 15/113, *aadA2*; 2/113 *kdpE*; 28/113), tetracycline resistance genes (*tetA*; 7/113, *tetB* ; 7/113, *tetC* ; 1/113, *emrK* ; 18/113, *emrY* ; 18/113, *mdfA* ; 20/113,), chloramphenicol resistance genes (*floR*; 2/113), trimethoprim/sulfamethoxazole resistance genes (*sul1*; 1/113, *sul2*; 10/113, *dfrA1*; 1/113, *dfrA5*; 4/113, *dfrA12*; 1/113, *dfrA16*; 1/113) and multi-drug efflux pump genes (*acrA, acrB, acrD*; 28/113, *tolC, baeR, emrA*; 10/113, *emrB*; 10/113) were all identified from WGS data. We identified 42 different sequence types (ST) covering 113 isolates where ST 10 was significant in 25 isolates followed by ST 1125 (10 isolates), ST 58 (8 isolates), ST 731 (6 isolates), ST 88 and 1121 (5 isolates). Of the 42 different STs, isolates from 16 STs showed resistance towards at least one antibiotic. More specifically, 36 % of the isolates from ST 10, 30 % from ST 1125, 50 % from ST 58, and 60 % from ST 88 showed either single/multi/extensive drug-resistance.

Genomic studies revealed the distribution of both acquired and intrinsic metal resistance genes among the isolates (Table 2). Acquired copper and silver resistant genes such as *pcoC, pcoE, copB, copD*, and *silE, silP* respectively were detected in 6 out of 113 isolates. Cationic efflux system protein genes such as *cusA, cusB, cusC, cusF, cusS*, *cusR* were detected in 98.23 % of the isolates. Intrinsic copper resistant genes such as *copA* and *cueO*, and zinc resistant genes such as *zntA, zntB, znuA, znuB, znuC, zitB, zraP* were identified in all the isolates.

### Efflux pump and ß-lactamase enzyme activities among the AMR isolates

**Fig. 3:**
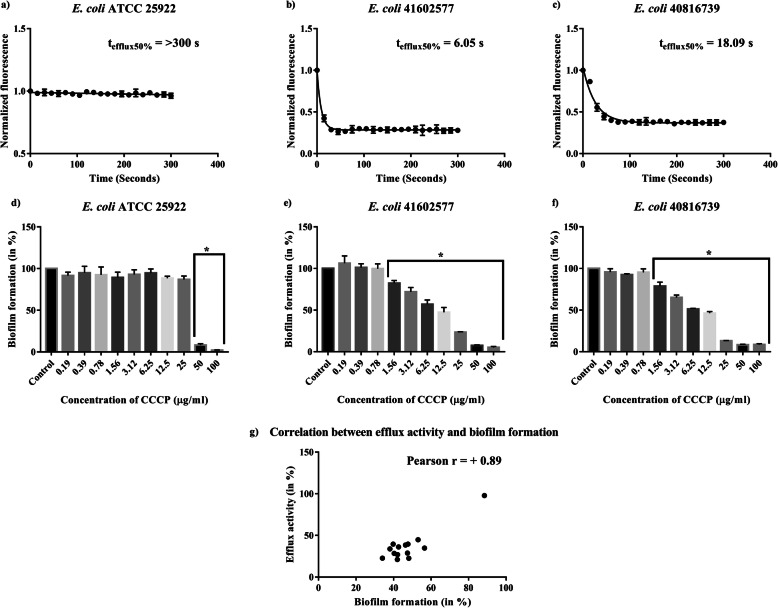
**Efflux-pump activity, and the impact of efflux inhibitor on biofilm formation.** Representative data on efflux activities of *E. coli* QC strain (**a**), isolate 41602577 (**b**), and isolate 40816739 (**c**). Nile red efflux assay was performed using 50 μM of CCCP and 10 μM of Nile red. The fluorescent intensity (544 nm/650 nm) of bacterial cells prior exposed to Nile red was monitored for 120 s before triggering the efflux pump by glucose addition. The fluorescence intensity was monitored for another 300 s. Likewise, a crystal violet assay was performed to assessthe relation between biofilm-forming ability and the efflux activity of the bacterial isolates (**d-f**). * indicates a significant decrease in biofilm formation when compared with the control. One-way ANOVA was performed to check the statistical significance of the obtained data where a p-value ≤ 0.05 was considered significant. **g**. Depicting a significant positive correlation (p<0.0001, Pearson r = +0.89) between efflux activity and biofilm-forming ability of the isolates. GraphPad Prism 7 software was used to perform the statistical analysis

We calculated the time required for the *E. coli* cells to extrude half of the probe molecule (Nile Red) and denoted it as t_efflux50 %_ (Table 1 and supplementary figure S1). Isolate 41602577 had the fastest extrusion (6.05 s), whereas isolate 40816739 had the slowest extrusion (18.09 s) (Fig. 3a-c).

**Fig. 4:**
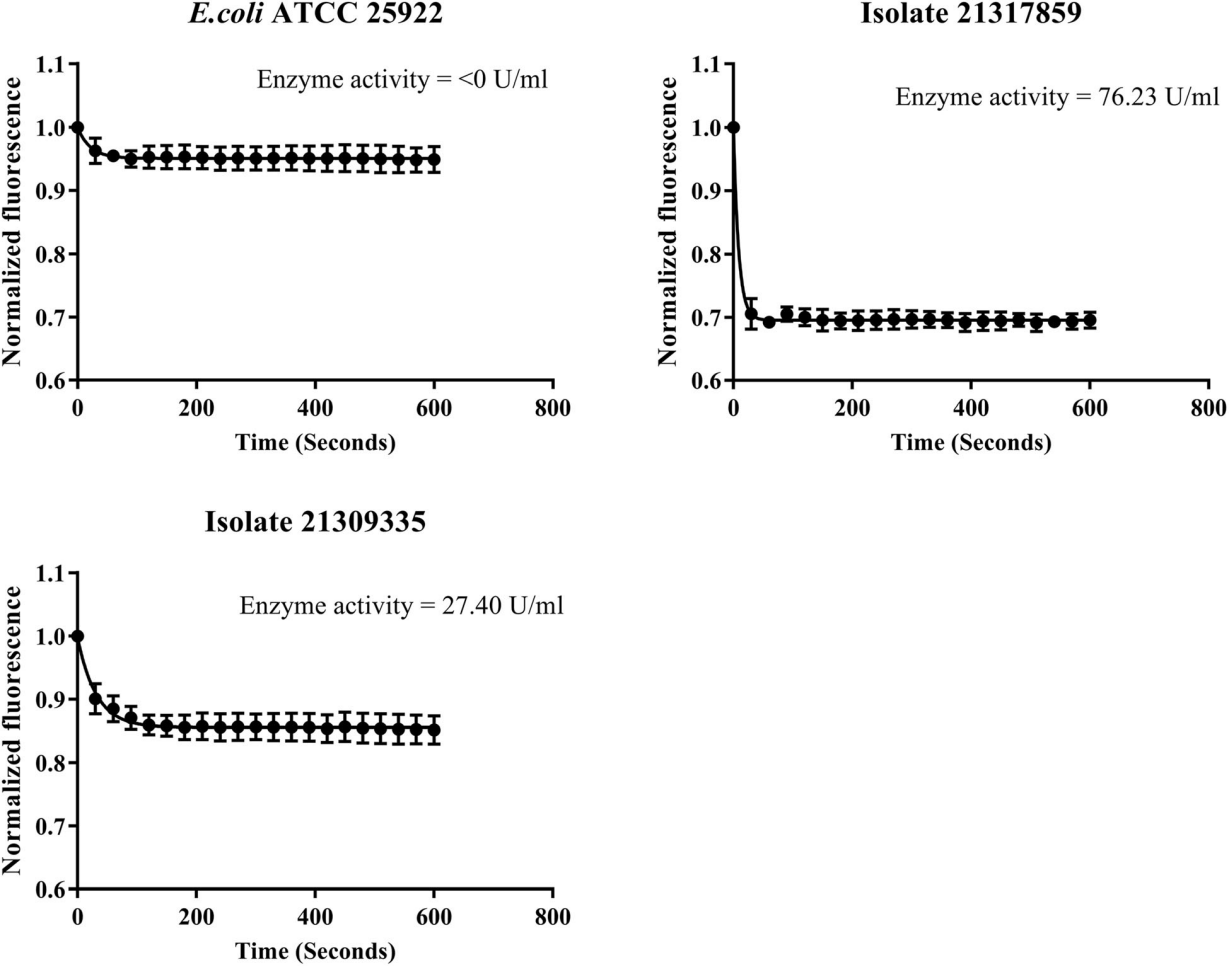
**ß-lactamase enzyme activity. **Bacterial cultures in MHB media were exposed to ampicillin and incubated under constant shaking. The cell suspensions were further washed and centrifuged. The cell-free extract was obtained and used as the source of ß-lactamase enzyme for the Nitrocefin assay. The absorbance of the cell-free extract mixed with nitrocefin and buffer solution was immediately detected in kinetic mode at 390 nm for 10 mins using a plate-reader. The ß-lactamase enzyme activity was calculated using the formula: ß-lactamase enzyme activity = {Sa/(Reaction time x Sv)}s

We detected 14 out of 32 AMR isolates exhibiting ß-lactamase enzyme activity (Table 1 and supplementary figure S2). Isolates 21317859 and 21309335 showed the highest (76.23 U/mL) and lowest (27.40 U/mL) enzyme activities, respectively (Fig. 4). Out of 14 isolates with ß-lactamase activity, 10 isolates were also identified with functional AcrAB-TolC efflux genes. Out of 14 isolates showing ß-lactamase activity, 42.85 % of them carried *bla*_TEM−1_, 14.28 % of the isolates carried *bla*_CMY−59_, and 7.14 % isolates had *bla*_CARB−3_. We observed a discrepancy between the phenotypic observations and WGS analysis as no particular gene was detected in 5 out of the 14 isolates exhibiting ß-lactamase activity.

### Production of hemolysis and correlation between efflux activity and biofilm formation

Out of 113 *E. coli* isolates, 33 isolates (29.20 %) produced the exotoxin α-hemolysin out of which 10 isolates were either single or multiple-antibiotic resistant (Table 3). *hlyE* was identified in all 113 isolates, whereas 32 isolates that produced α-hemolysin had *hlyA, hlyB, hlyC*, and *hlyD* (Table 3 and supplementary table S6.b.).

**Table 3:**
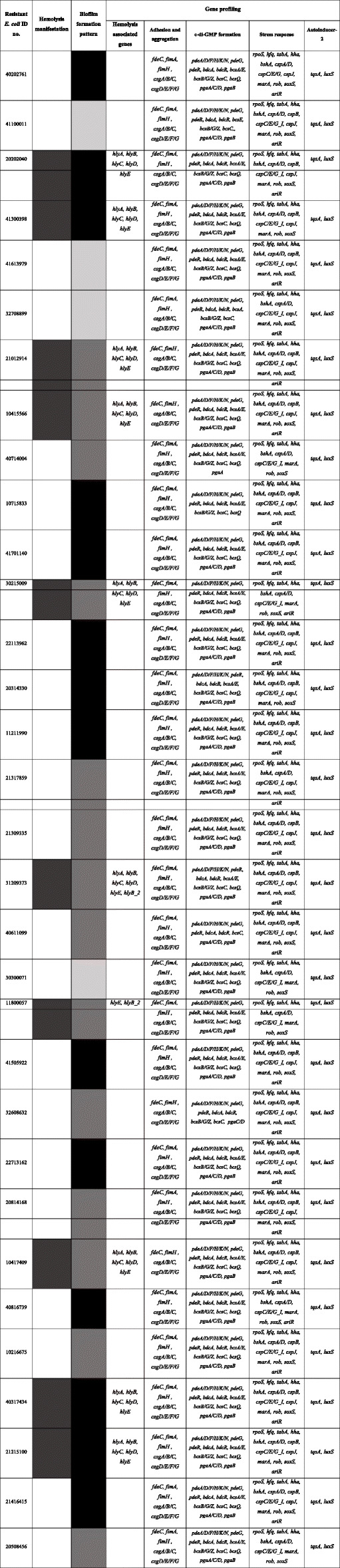
Patterns associated with the virulence factors and gene profile of the 32 antibiotic-resistant *E. coli* isolates

Color codes: Weak biofilm-formers-Light Grey; Moderate biofilm formers-Dark Grey; Strong biofilm formers-Black; Hemolysis manifestation-Light Black

**Fig. 5:**
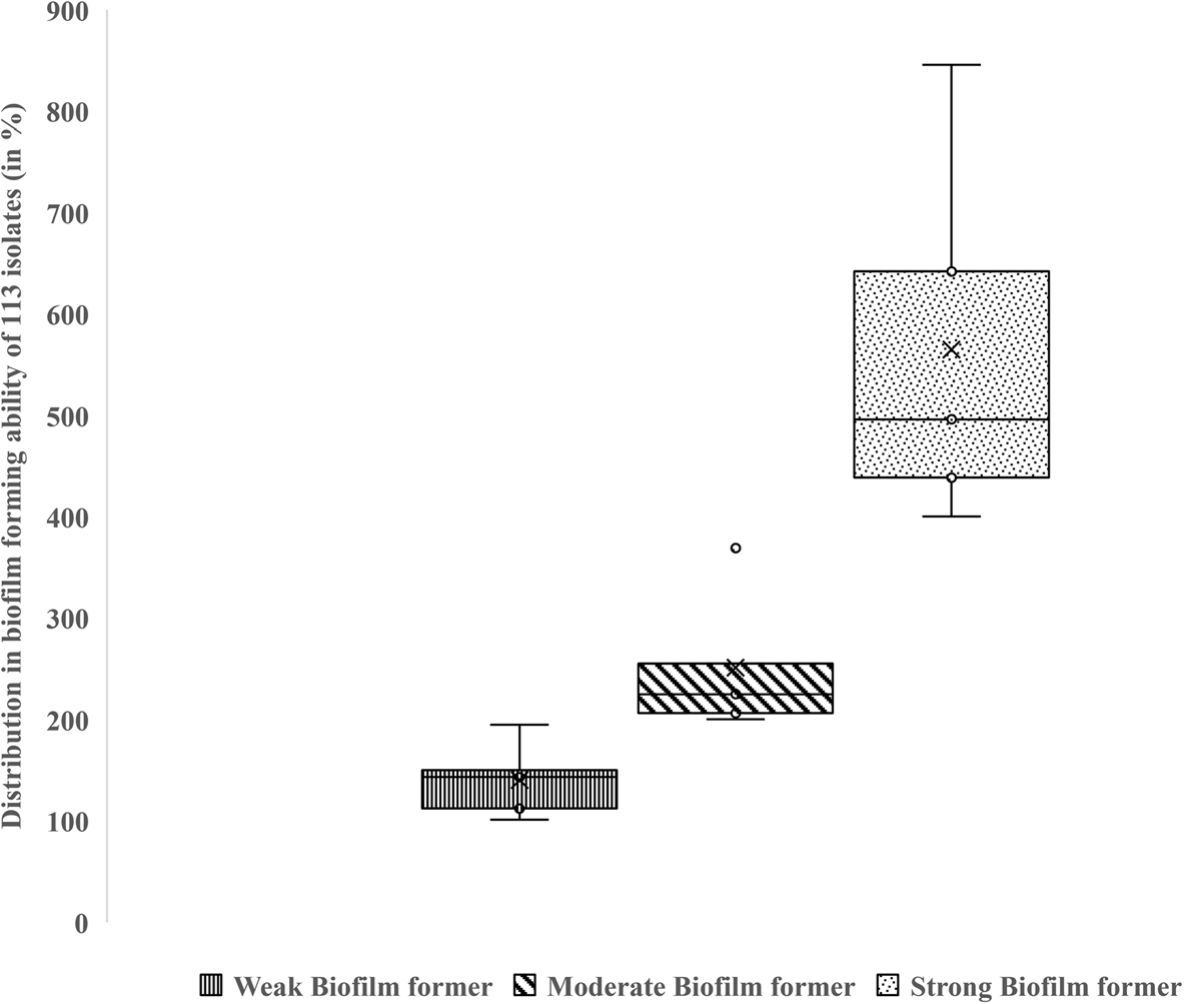
**Distribution and diversity biofilm formation for 113** ***E. coli*** **isolates.** Bacterial cultures were normalized to 0.5 McFarland standard and added to MH broth. The plates were incubated without shaking. The biofilm formation was assessed using crystal violet assay and the levels of biofilm formation were categorized based on OD. The biofilm-forming ability was further classified as: Biofilm breakpoint (%) ≤ 100% = Non-biofilm formers (NBF), 100% < Biofilm breakpoint (%) ≤ 200% = Weak Biofilm formers (WBF), 200% < Biofilm breakpoint (%) ≤ 400% = Moderate Biofilm formers (MBF), Biofilm breakpoint (%) > 400% = Strong Biofilm formers (SBF)

We detected biofilm-forming ability in all 113 *E. coli* isolates (supplementary table S6.b.). Specifically, 19.46 % of the isolates were observed to be strong biofilm formers, whereas 49.55 % of them were moderate biofilm formers and 30.99 % of the isolates were weak biofilm formers (Fig. 5). We didn’t find any conclusive correlation between the mastitis severity scores and the biofilm-forming ability of the isolates. However, 51.4 % (out of 35) and 47.7 % (out of 65) of the isolates from mastitis scores 2 and 3 respectively formed moderate biofilms, whereas 17.1 % (from mastitis score 2) and 18.5 % (from mastitis score 3) formed strong biofilms.

All antibiotic-resistant isolates (n = 32) were either moderate (n = 18) or strong (n = 14) biofilm formers (Table 3). Genomic characterization revealed the presence of several genes that are responsible for adhesion, aggregation, c-di-GMP formation, stress response, and autoinducer-2 quorum sensing (Table 3).

We also investigated a possible relationship between efflux pump activity and the biofilm-forming ability of *E. coli*. The biofilm formation of all the 13 isolates with functional efflux pump was significantly lowered (p < 0.05) when they were subjected to the efflux-pump inhibitor, CCCP, while the biofilm-forming ability of the QC strain (without efflux pump activity) wasn’t affected by CCCP (supplementary figure S3 and S4). Figure 3d-f shows the impact of CCCP on the biofilm-forming ability of isolate 41602577 (with the fastest extrusion), isolate 40,816,739 (with the slowest extrusion), and QC strain (with non-functional AcrAB-TolC). The efflux activity showed a significant positive correlation (p < 0.0001, Pearson r = + 0.89) with the biofilm-forming ability of the 13 isolates (Fig. 3g).

## Discussion

In this study, we evaluated the prevalence of AMR in *E. coli* isolates from the cases of clinical bovine mastitis in Canada. Several strains showed resistance towards one or more antibiotics and metals. Interestingly, the study found that irrespective of the non-resistant responses by many *E. coli* isolates towards antibiotics could still possess metal resistance properties and virulence characteristics. Further investigation identified efflux pump activity and ß-lactamases along with corresponding genes (ß-lactamase producing genes: *bla*_TEM−1_, *bla*_CARB−3_, *bla*_CMY−59_, efflux pump inducing genes: *acrA, acrB, acrD, tolC, baeR, emrA, emrB*). Apart from AMR properties, we also found virulence factors such as biofilm formation and hemolysis and associated genes in several isolates that support bacterial survival in host tissues. Notably, there was a positive correlation between efflux pump activity and biofilm formation.

Of the 113 isolates included in this study, 28.31 % were shown to be resistant to at least one antibiotic. The rate of resistance seen in our study was comparable with previous studies that had examined a larger library of *E. coli* isolates from bovine mastitis [15]. All isolates showed susceptibility towards ciprofloxacin and ofloxacin, which was in agreement with earlier observations [15, 16]. The effectiveness of these antibiotics was possibly due to their less frequent application in Canadian dairy farms. In Canada, the use of these antibiotics has been restricted for farm applications to minimize the chance of resistance emergence against these last-resort drugs for human applications [17].

Although antimicrobial susceptibility testing for Canadian *E. coli* isolates from cases of bovine mastitis has been performed in the past, this study went on to identify the genes that confer AMR including the ones that are transmissible through horizontal gene transfer [4]. Out of the fourteen isolates with β-lactamase enzyme activity, two isolates carried *bla*_CMY−59_, three isolates carried *bla*_TEM−1B_, one carried *bla*_CARB−3_. This was one of a few cases that identified *cmy* and *tem* genes in the isolates from Holstein dairy cattle among other two studies which identified these genes in *E. coli* isolates from colostrum and feces of the cattle in New Brunswick [10, 18]. Other important emerging resistance genes found in our study included tetracycline resistance genes (*tetA, tetB, tetC*) and aminoglycoside resistance gene (*aadA2*) which were not identified from any isolates from CM by the previous studies although the phenotypic resistance to corresponding drugs was identified [4].

The isolates 10,800,294 and 21,914,232 showed resistance to cefazolin and cefotaxime without ESBL or plasmid-mediated AmpC β-lactamase genes. The expression of their β-lactamase enzyme activities was less than that of other isolates that had *bla*_CMY−59_, *bla*_TEM−1B_, and *bla*_CARB−3_. Their resistance might be due to extrusion by efflux pump and biofilm-forming ability: Isolate 10,800,294 was a strong biofilm former and had an active AcrAB-TolC, whereas isolate 21914232 was a moderate biofilm former (t_efflux50 %_=11.35 s) [19, 20]. Interestingly, two other isolates, 40611099 and 31801812 showed resistance to colistin while none of them harbored MCR genes and plasmid-mediated colistin determinants genes. This also might be due to complex mechanisms by efflux pump in the case of isolate 31801812 which had a strong efflux pump activity (t_efflux50 %_=7.03 s) [21]. Therefore, despite the ESBL, plasmid-mediated AmpC β-lactamase, and MCR as emerging resistance, the assessment of the efflux pump mediated resistance to clinically important drugs such as β-lactams and colistin is required for a better understanding of AMR emergence and its potential increase in dairy farms. Isolates 40611099 and 21914232 had no AcrAB-TolC efflux activity and it might employ several other previously reported strategies against polymixins including a variety of lipopolysaccharides (LPS) modifications, such as modifications of lipid A with phosphoethanolamine and 4-amino-4-deoxy-L-arabinose, and overexpression of the outer membrane of protein OprH [21]. Ampicillins and cephalosporins resistant isolates without any acquired β-lactamase genes could be because of the mutations in the promoter regions of the chromosomal *E. coli* AmpC gene [22]. Efflux or ß-lactamase enzyme activities were not identified in 15 of the 32 AMR isolates. The existence of alternate resistant mechanisms such as limiting hydrophilic drug uptake or drug-target modifications *via* the acquisition of the plasmids carrying 16S rRNA methyltransferases and other enzymes could be the possible reasons [23].

We observed 33 isolates with hemolysin activity. Of the hemolytic isolates, 10 were also resistant to one or more antibiotics. The hemolysin phenotype corresponded with the presence of genetic determines *HlyA/E/C/B/D*, which were also identified in our genomic analysis. α-hemolysis is an important secretory virulence factor that is reported to be produced by 20–50 % of strains from bovine IMI [3].

The *E. coli* isolates produced biofilms, that included weak (n = 35), moderate (n = 56), and strong (n = 22) biofilm formers. Different sets of genes that confer biofilm formation were identified which encode adhesion, aggregation, c-di-GMP formation, stress inducer, and autoinducer-2. The potential contributions of *csgB/A* and *csgD/E/F/G* as a host cell adhesion and invasion mediator, and inducers of the host inflammatory responses; *pde, bdc, bcs*, and *pga* gene involvement in chemotaxis, surface colonization, and persistence have already been established [24, 25]. The transcription factors; *marA, soxS*, and *rob* found in our study are reported to play a crucial role in mediating MDR by up-regulating the expression of the AcrAB-TolC efflux pump [26].

Efflux systems have been established to be a contributing factor in the intrinsic antibiotic resistance by *E. coli* [27]. The decreased biofilm formation in the 13 AMR isolates by inhibiting their efflux activity showed a possible role of efflux pump in *E. coli* biofilm formation. Generally, four possible roles of efflux pumps in biofilm formation are postulated: indirect regulation of genes involved in biofilm formation, efflux of extra-polymeric substances/quorum sensing (QS) and quorum quenching molecules to facilitate biofilm matrix formation and regulate QS respectively, efflux of threatening antibiotics and metabolic intermediates, promote aggregation or prevent adhesion to surfaces and other cells [28]. The QseBC regulator found in our study has previously been reported to upregulate the transcription of the efflux-pump-associated genes in *E. coli* isolated from mastitis cases [27].

Of the 113 isolates, 107 of them were resistant to at least one metal tested. The antibacterial efficiency of copper and zinc against *E. coli* isolates identified in our study contradicts significantly by more than 80 and 60-folds respectively, from a study reported by Hoque et al. with *E. coli* isolates from mastitis cases of Bangladesh [2]. However, less is known about the use of these heavy metals in Canadian dairy cow feed and their content in raw milk [29]. Therefore, it is difficult to identify the significance of the metal-resistant *E. coli* from bovine mastitis which requires more investigations on the use of heavy metals to correlate with its resistance. The identified copper and silver resistant genes such as *pcoC, pcoE, copB, copD* and *silE, silP* respectively and cationic efflux system proteins such as CusA, CusB, CusC, CusF, CusS, CusR in our study are previously reported to be involved in the detoxification of copper and silver in *E. coli* as a part of the CusCFBA copper/silver efflux system [30]. Genes such as *zntA, zntB, znuA, znuB, znuC, zitB, zraP* identified in the *E. coli* chromosome are also reported to be one of the key factors for zinc resistance [31].

## Conclusions

Unlike other pathogens, intramammary infections caused by *E. coli* rarely require antibiotic interventions but are reported to cause persistent infection [5]. Given the possibility of shedding of *E. coli* in milk and AMR transmittance to other pathogenic bacteria, the finding that resident *E. coli* harbors multiple/extensive drug resistance and virulence characteristics have implications for public health. Further, unveiling prevalent mechanisms of AMR in pathogenic bacteria from animal farms is vital for designing novel drugs and treatment strategies. Results from our study suggest the inadequacy of antimicrobials with a single mode of action to curtail AMR bacteria with multiple mechanisms of resistance and virulence factors and therefore, calls for combinatorial-therapy for effective management of AMR infections in dairy farms and combat its potential transmission to the food supply chain through the milk and dairy products. As biofilm formation and efflux activity play a major role in the persistence of bacteria in bovine udders and resistance towards several antimicrobials, the relation between efflux property and biofilm-forming ability is shown in our study would possibly open up a new horizon in the development of combinatorial-therapeutic strategies.

## Methods

### Isolation of the *E. coli* isolates from cases of clinical mastitis

*E. coli* isolates used in this study were a part of the mastitis pathogen culture collection (MPCC) across Alberta, Ontario, Quebec, and Atlantic provinces (Prince Edward Island, Nova Scotia, and New Brunswick) [12]. Each isolate was obtained as previously described [4, 32]. The metadata including number and location of the herd, cow ID, quarter position, sampling date, mastitis severity score, days in milk (DIM) at sampling, and cow’s parity is summarized in Supplementary table S1 [33].

Single colonies of 113 bacterial isolates grown in Tryptic Soy Agar (TSA) plates containing 5 % sheep blood agar (Hardy Diagnostics, Canada) was inoculated in Mueller-Hinton broth (MHB) (Millipore Sigma, Canada) and kept for incubation at 37 °C under shaking (4 x g) for 18 h for obtaining freshly grown bacterial cells for conducting assays.

### Susceptibility testing of *E. coli* isolates against a panel of antibiotics

The *E. coli* isolates were subjected to Kirby-Bauer disk diffusion susceptibility tests following the protocol in the Clinical and laboratory standard institute (CLSI) guidelines [34]. Eighteen antibiotics (Oxoid, Thermo Fischer Scientific, Canada) relevant to human and animal health from the classes of ß-lactams, aminoglycosides, cephalosporins, quinolones, tetracycline, chloramphenicol, sulphonamide, and polymyxin were included in this study. The list of antibiotics tested and their corresponding MIC values are given in supplementary table S2.a. *E. coli* ATCC 25,922, *S. aureus* ATCC 25,923, and *P. aeruginosa* ATCC 27,853 (Oxoid company, Canada) were used as the quality control (QC) strains. As previously described, the isolates were labeled as multidrug-resistant (non-susceptible to ≥ 1 antibiotic in ≥ 3 antibiotic classes), extensively drug-resistant (non-susceptible to ≥ 1 antibiotic in all but ≤ 2 antibiotic classes), and single drug-resistant (non-susceptible to 1 antibiotic) based on their responses towards the selected antibiotic classes [35].

### Susceptibility testing of *E. coli* isolates against heavy metals

The sensitivities of the *E. coli* isolates to metals were assessed using the broth microdilution method as previously reported [2]. Three metal salts viz. copper sulfate (CuSO_4_), zinc sulfate (ZnSO_4_), and silver nitrate (AgNO_3_) were used in this assay. Ten-twofold serial dilutions of metal salts were prepared in 100 µL of autoclaved Mueller-Hinton broth (MHB) (Millipore Sigma, Canada) in a 96 well plate (Millipore Sigma, Canada) wherein the final concentrations were 5, 5, and 2 mg/mL for CuSO_4_, ZnSO_4_, and AgNO_3_, respectively. Wells in these plates were added with 10 µL of freshly prepared bacterial culture in MHB adjusted to 0.5 McFarland standard. *E. coli* ATCC 25922 was used as the quality control (QC) strain. These 96 well plates were incubated for 18 h at 37 °C in a shaking incubator.

The bacterial viability was monitored by resazurin assay [36]. Briefly, 30 µL of resazurin solution (0.5 % in PBS) was added to each of the wells and further incubated for 2 h at 37 °C under shaking. The fluorescent intensity (530 nm for excitation and 590 nm for emission) was measured using a plate reader (SpectraMax-i3X, Molecular Devices, USA).

Background corrected fluorescence intensity data were used to generate a dose-response curve. The inhibitory concentration (50 %) or IC_50_ values of each metal salts against each *E. coli* isolate were calculated using GraphPad Prism 7 software where IC_50_ is the ability of the metal salts to inhibit 50 % of bacterial growth. The IC_50_ value of each metal salt against the QC strain was considered as the cut-off concentration. *E. coli* isolates with IC_50_ values less or equal or non-significant (p > 0.05) to than that of the cut-off were considered as susceptible, whereas significant (p ≤ 0.05) non-susceptible isolates were categorized into weakly resistant isolates (WRI) (QCIC_50cut − off_ < WRI ≤ 1.5 folds of QCIC_50cut − off_ ), moderately resistant isolates (MRI) (1.5 folds of QCIC_50cut − off_ < MRI ≤ 2 folds of QCIC_50cut − off_) and strongly resistant isolates (SRI) (SRI > 2 folds of QCIC_50cut − off_).

### Assessing efflux pump activity in antibiotic-resistant *E. coli* isolates

Quantification of efflux pump activity in the AMR *E. coli* isolates was carried out by Nile red efflux assay as previously described [37]. Briefly, 1 mL of bacterial cells in MHB was centrifuged at 2,300 x g for 10 min at room temperature (RT). The supernatant was discarded, and the cell pellet was re-suspended with 20 mM potassium phosphate buffer (pH 7) containing 1 mM MgCl_2_ (PPB). Cells washed and suspended in PPB (1.0 McFarland standard) in glass test tubes were added with carbonyl cyanide 3-chlorophenylhydrazone (CCCP) (50 µM) and incubated for another 15 min at RT. Subsequently, Nile red (10 µM) (dissolved in 10 % dimethyl formamide-90 % ethanol (v/v)) was added to each of the tubes, incubated for 2 h at 37 °C under shaking, and then kept at RT for an hour. After incubation, the cell suspensions were centrifuged, washed twice, and resuspended in PPB. The suspension (140 µL) was transferred to the wells of the 96 well plate. The fluorescent intensity (544 nm for excitation and 650 nm for emission) was monitored for 120 s using the plate reader. Nile red efflux was triggered by rapid energization with 10 µL of glucose (25 mM) and fluorescence was monitored for another 300 s. PPB without cell suspension was used as blank and *E. coli* ATCC 25,922 was used as a control.

Data from the experiments were plotted using GraphPad Prism 7. Time-dependent efflux of Nile red was fitted using a single exponential decay equation:

$$\mathrm Y\;=\;\left({\mathrm Y}_\circ-\;\mathrm{Plateau}\right)\;\times\;\exp\left(-\mathrm K\;\times\;\mathrm X\right)\;+\;\mathrm{Plateau}$$

where Y˳ is the Y value when X (time) is zero, the plateau is the Y value at infinite times and K is the rate constant. Efflux was initiated at t = 0 by energization with glucose and reached 50 % complete at t_efflux50 %_. The equation was used to calculate the t_efflux50 %_ which indicates the time required for the *E. coli* cells to extrude half of the preloaded Nile red molecules.

### Detection of ß-lactamase activity in antibiotic-resistant *E. coli* isolates

Bacterial isolates grown for 18 h in MHB were used for preparing 1.0 McFarland standard in 1 mL of fresh MHB. Ampicillin (50 µg/mL) was added to each of the cell suspensions and incubated for 3 h at 37 °C under constant shaking. After incubation, the cell suspensions were centrifuged at 8,900 x g for 10 min, suspended in sodium phosphate buffer (pH7.0), and washed. The suspensions were resuspended again in the buffer, sonicated for 3 min in the presence of ice, and centrifuged at 17,500 x g for 25 min to obtain the cell-free extract, which was used as the source of ß-lactamase enzyme for Nitrocefin assay as detailed previously [38, 39]. Briefly, 10 µL of nitrocefin (Abcam, Canada), a chromogenic cephalosporin dissolved in 5 % DMSO (stock concentration of 0.5 mg/mL), was mixed with 10 µL of the cell-free extract and the volume was adjusted to 100 µL using buffer solution in a 96-well plate. The absorbance was immediately detected in kinetic mode at 390 nm for 10 min using a plate-reader. Nitrocefin added to buffer solution without cell-free extract and *E. coli* ATCC 25922 was used as a media and negative control, respectively.

A nitrocefin standard curve (concentration ranging from 125 µg/mL to 0.49 µg/mL) was plotted against absorbance (390 nm). The ß-lactamase enzyme activity was calculated using the formula: ß-lactamase enzyme activity = {S_a_/(Reaction time x S_v_)}s.

where, S_a_ is the amount of Nitrocefin (in µM) hydrolyzed in the unknown sample well between T1 and T2 of the standard curve, Reaction time is the difference between absorbance detected in two-time intervals (T1 and T2 in minutes), S_v_ is the sample volume (in mL) added to the well. ß-lactamase activity is reported as U/mL.

### Assessing virulence factors and evaluating the relationship between efflux activity and biofilm-formation in AMR isolates

Detection of hemolysis was carried out as previously reported [40]. A loopful of *E. coli* from agar plates was inoculated into 10 mL of sterile TSB media and incubated overnight. The isolates were then streaked in Tryptic Soy Agar (TSA) plates containing 5 % sheep blood. The pattern of hemolysis was detected by visual inspection for the translucency around the bacterial colony that occurs due to the lysis of red blood cells.

The biofilm-forming ability was assessed by crystal violet assay [36]. Briefly, 100 µL of autoclaved MH broth was transferred to each of the wells of a 96 well plate and 10 µL of the bacterial culture maintained at 0.5 McFarland standard was added to each of the wells. The plates were incubated for 24 h at 37 °C without shaking. After 24 h of incubation, the media was removed from the wells and washed twice with pre-autoclaved saline to remove non-adherent cells. A 100 µL of 99 % methanol was added to each well to fix the biofilms and kept undisturbed for 15 min at room temperature. The wells were further washed with saline and air-dried and added with 200 µL of crystal violet (0.4 %) and left undisturbed for 2 h. The wells were again washed with saline, air-dried followed by the addition of 30 % acetic acid. The absorbance was detected at 570 nm using a plate reader.

The classification of the biofilm-forming ability of *E. coli* isolates was obtained by using the following formula as previously mentioned by Hoque et al.: OD_cut−off_ = OD_avg_ of control + 3 x standard deviation (SD) of ODs of control; OD ≤ OD_cut−off_ = Non-biofilm-former (NBF); OD_cut−off_ < OD ≤ 2 × OD_cut−off_ = Weak biofilm-former (WBF); 2 × OD_cut−off_ < OD ≤ 4 × OD_cut−off_ = Moderate biofilm-former (MBF); OD > 4 × OD_cut−off_ = Strong biofilm-former (SBF) [2]. A similar assay was performed with a concentration range of CCCP (from 100 µg/mL to 0.19 µg/mL) to assess the relation between biofilm-forming ability and efflux activity of the bacterial isolates. Media with bacteria but no efflux inhibitor were included as a negative control, and wells without bacteria and efflux inhibitor were included as media controls. *E. coli* ATCC 25,922 was used as a control strain to check the difference in biofilm formation. Pearson correlation test was performed between efflux activity of each isolate at a saturation point (considering 180 s after re-energization) and the biofilm-forming capacity of the corresponding isolates at 50 µM of CCCP. The Pearson’s correlation and One-way ANOVA (p-value ≤ 0.05 was regarded as significant) tests were performed using GraphPad Prism 7 software. Irrespective of the *E. coli* isolates, the biofilm inhibitory concentrations below the MIC of CCCP (checked at OD_600_) were considered as the concentrations of interest to demonstrate an antibiofilm effect rather than a generalized growth inhibition [41].

### Identification of sequence type, antibiotic, and metal resistance genes

Extraction and quantification of DNA of each isolate, DNA library preparation, whole-genome sequencing, assembly, and annotation of sequenced reads were conducted as previously described (supplementary table S3**)** [13, 14]. Assembly was conducted using ProkaryoteAssembly version 0.1.6 (https://github.com/bfssi-forest-dussault/ProkaryoteAssembly) [42–44]. The coverage and the number of contigs were identified and the contigs shorter than 1 kbp were discarded using Qualimap, whereas Prokka was used to annotate the assembled reads [45, 46].

Sequence types (STs) of each isolate were identified using the tool most (https://github.com/tseemann/mlst) which incorporates data from the PubMLST database [47]. Antibiotic resistance genes were identified by Prokka and ABRicate v1.0 (https://github.com/tseemann/abricate) with CARD and ResFinder databases [46]. Metal resistance genes were identified by Prokka v.1.14.5 and ABRicate with MEGAres database [46, 48]. Minimum coverage and identity settings for all the screening was set to 90 %.

## Supplementary Information

**Additional file 1.**

**Additional file 2.**

## Data Availability

All supporting datasets have been deposited online. Whole genome sequencing data were deposited in BioProject PRJNA612640 and the accession numbers for each genome are reported in Table S3.

## References

[1] Aghamohammadi M, Haine D, Kelton DF, Barkema HW, Hogeveen H, Keefe GP, et al. Herd-level mastitis-associated costs on Canadian dairy farms. Front Vet Sci. 2018;5(100). 10.3389/fvets.2018.00100. 10.3389/fvets.2018.00100 PMC5961536 29868620

[2] Hoque MN, Istiaq A, Clement RA, Gibson KM, Saha O, Islam OK, et al. Insights into the resistome of bovine clinical mastitis microbiome, a key factor in disease complication. Front Microbiol. 2020;11(860). 10.3389/fmicb.2020.00860. 10.3389/fmicb.2020.00860 PMC7283587 32582039

[3] Dego OK. Bovine mastitis: part I. In: Animal reproduction in veterinary medicine. IntechOpen; 2020. https://www.intechopen.com/online-first/bovine-mastitis-part-i, 10.5772/intechopen.93483. Accessed 03 Mar 2021.

[4] Fairbrother JH, Dufour S, Fairbrother JM, Francoz D, Nadeau E, Messier S (2015). Characterization of persistent and transient *Escherichia coli* isolates recovered from clinical mastitis episodes in dairy cows. Vet Microbiol.

[5] Bradley A, Green M (2001). Adaptation of *Escherichia coli* to the bovine mammary gland. J Clin Microbiol.

[6] Saini V, McClure JT, Léger D, Dufour S, Sheldon AG, Scholl DT (2012). Antimicrobial use on Canadian dairy farms. J Dairy Sci.

[7] Argudín MA, Hoefer A, Butaye P (2019). Heavy metal resistance in bacteria from animals. Res Vet Sci.

[8] Catry B, Laevens H, Devriese LA, Opsomer G, De Kruif A (2003). Antimicrobial resistance in livestock. J Vet Pharmacol Ther.

[9] Piddock LJ (1996). Does the use of antimicrobial agents in veterinary medicine and animal husbandry select antibiotic-resistant bacteria that infect man and compromise antimicrobial chemotherapy?. J Antimicrob Chemother.

[10] Awosile BB, McClure JT, Sanchez J, VanLeeuwen J, Rodriguez-Lecompte JC, Keefe G (2017). Short communication: Extended-spectrum cephalosporin-resistant *Escherichia coli* in colostrum from New Brunswick, Canada, dairy cows harbor blaCMY-2 and blaTEM resistance genes. J Dairy Sci.

[11] Bohnert JA, Karamian B, Nikaido H (2010). Optimized Nile Red efflux assay of AcrAB-TolC multidrug efflux system shows competition between substrates. Antimicrob Agents Chemother.

[12] Reyher KK, Dufour S, Barkema HW, Des Côteaux L, DeVries TJ, Dohoo IR (2011). The National Cohort of Dairy Farms—A data collection platform for mastitis research in Canada. J Dairy Sci.

[13] Jung D, Park S, Ruffini J, Dufour S, Ronholm J (2021). Draft genome sequences of 113 *Escherichia coli* strains isolated from intramammary infections in dairy cattle. Microbiol Resour Announcement.

[14] Dongyun Jung SP, Janina Ruffini, Forest Dussault, Simon Dufour, Jennifer Ronholm. Comparative genomic analysis of *Escherichia coli* isolates from cases of bovine clinical mastitis identifies nine specific pathotype marker genes. Microbial Genomics-‘Accepted’. 2021. 10.1099/mgen.0.000597 PMC8477405 34227932

[15] Saini V, McClure JT, Léger D, Keefe GP, Scholl DT, Morck DW (2012). Antimicrobial resistance profiles of common mastitis pathogens on Canadian dairy farms. J Dairy Sci.

[16] Makovec JA, Ruegg DPL (2003). Antimicrobial resistance of bacteria isolated from dairy cow milk samples submitted for bacterial culture: 8,905 samples (1994–2001). J Am Vet Med Assoc.

[17] Uses of Antimicrobials in Food Animals in Canada: Impact on Resistance and Human Health In. Edited by Veterinary Drugs Directorate HC; 2002. Link: https://www.canada.ca/en/health-canada/services/drugs-health-products/reports-publications/veterinary-drugs/uses-antimicrobials-food-animals-canada-impact-resistance-human-health-health-canada-2002.html. Accessed 03 Mar 2021.

[18] Awosile B, McClure J, Sanchez J, Rodriguez-Lecompte JC, Keefe G, Heider LC (2018). Salmonella enterica and extended-spectrum cephalosporin-resistant *Escherichia coli* recovered from Holstein dairy calves from 8 farms in New Brunswick, Canada. J Dairy Sci.

[19] Amanatidou E, Matthews AC, Kuhlicke U, Neu TR, McEvoy JP, Raymond B (2019). Biofilms facilitate cheating and social exploitation of β-lactam resistance in *Escherichia coli*. NPJ Biofilms Microbiomes.

[20] Nishino K, Yamada J, Hirakawa H, Hirata T, Yamaguchi A (2003). Roles of TolC-dependent multidrug transporters of *Escherichia coli* in resistance to β-lactams. Antimicrob Agents Chemother.

[21] Olaitan AO, Morand S, Rolain J-M. Mechanisms of polymyxin resistance: acquired and intrinsic resistance in bacteria. Front Microbiol. 2014;5(643). 10.3389/fmicb.2014.00643. 10.3389/fmicb.2014.00643 PMC4244539 25505462

[22] Davis MA, Besser TE, Orfe LH, Baker KNK, Lanier AS, Broschat SL (2011). Genotypic-phenotypic discrepancies between antibiotic resistance characteristics of *Escherichia coli* isolates from calves in management settings with high and low antibiotic use. Appl Environ Microbiol.

[23] Reygaert WC (2018). An overview of the antimicrobial resistance mechanisms of bacteria. AIMS Microbiol.

[24] Barnhart MM, Chapman MR (2006). Curli biogenesis and function. Annu Rev Microbiol.

[25] Reinders A, Hee C-S, Ozaki S, Mazur A, Boehm A, Schirmer T (2016). Expression and genetic activation of cyclic Di-GMP-specific phosphodiesterases in *Escherichia coli*. J Bacteriol.

[26] Duval V, Lister IM (2013). MarA, SoxS and Rob of *Escherichia coli* - Global regulators of multidrug resistance, virulence and stress response. Int J Biotechnol Wellness Ind.

[27] Li W, Xue M, Yu L, Qi K, Ni J, Chen X (2020). QseBC is involved in the biofilm formation and antibiotic resistance in *Escherichia coli* isolated from bovine mastitis. PeerJ.

[28] Alav I, Sutton JM, Rahman KM (2018). Role of bacterial efflux pumps in biofilm formation. J Antimicrob Chemother.

[29] Zwierzchowski G, Ametaj BN. Mineral elements in the raw milk of several dairy farms in the province of Alberta. Foods. 2019;8(8). 10.3390/foods8080345. 10.3390/foods8080345 PMC6722752 31416263

[30] Franke S, Grass G, Rensing C, Nies DH (2003). Molecular analysis of the copper-transporting efflux system CusCFBA of *Escherichia coli*. J Bacteriol.

[31] Rensing C, Mitra B, Rosen BP. The zntA gene of *Escherichia coli* encodes a Zn (II)-translocating P-type ATPase. Proc Nati Acad Sci. 1997;94(26):14326-31. 10.1073/pnas.94.26.14326 10.1073/pnas.94.26.14326 PMC24962 9405611

[32] Dufour S, Labrie J, Jacques M (2019). The mastitis pathogens culture collection. Microbiol Resour Announcement.

[33] Sears PM, McCarthy KK. Diagnosis of mastitis for therapy decisions. Vet Clin North Am. 2003;19(1):93–108, vi. 10.1016/s0749-0720(02)00074-9. 10.1016/s0749-0720(02)00074-9 12682937

[34] CLSI (2017). Performance standards for antimicrobial susceptibility testing; CLSI document M100–S27.

[35] Magiorakos AP, Srinivasan A, Carey RB, Carmeli Y, Falagas ME, Giske CG, Harbarth S, Hindler JF, Kahlmeter G, Olsson-Liljequist B, Paterson DL, Rice LB, Stelling J, Struelens MJ, Vatopoulos A, Weber JT, Monnet DL (2012). Multidrug-resistant, extensively drug-resistant and pandrug-resistant bacteria: an international expert proposal for interim standard definitions for acquired resistance. Clin Microbiol Infect.

[36] George S, Teo LL, Majumder S, Chew WL, Khoo GH. Low levels of silver in food packaging materials may have no functional advantage, instead enhance microbial spoilage of food through hormetic effect. Food Control. 2020:107768. 10.1016/j.foodcont.2020.107768.

[37] Iyer R, Ferrari A, Rijnbrand R, Erwin AL (2015). A fluorescent microplate assay quantifies bacterial efflux and demonstrates two distinct compound binding sites in AcrB. Antimicrob Agents Chemother.

[38] Dai X, Xiang S, Li J, Gao Q, Yang K (2012). Development of a colorimetric assay for rapid quantitative measurement of clavulanic acid in microbial samples. Sci China Life Sci.

[39] Sharma S, Ramnani P, Virdi JS (2004). Detection and assay of β-lactamases in clinical and non-clinical strains of *Yersinia enterocolitica biovar* 1A. J Antimicrob Chemother.

[40] Buxton R. Blood agar plates and hemolysis protocols. Am Soc Microbiol. 2005. Link: https://asm.org/getattachment/7ec0de2b-bb16-4f6e-ba07-2aea25a43e76/protocol-2885.pdf. Accessed 03 Mar 2021.

[41] Baugh S, Phillips CR, Ekanayaka AS, Piddock LJV, Webber MA (2013). Inhibition of multidrug efflux as a strategy to prevent biofilm formation. J Antimicrob Chemother.

[42] Brian B. BBTools software package. In.; 2014.

[43] Souvorov A, Agarwala R, Lipman DJ (2018). SKESA: strategic k-mer extension for scrupulous assemblies. Genome Biol.

[44] Walker BJ, Abeel T, Shea T, Priest M, Abouelliel A, Sakthikumar S (2014). Pilon: an integrated tool for comprehensive microbial variant detection and genome assembly improvement. PLOS ONE.

[45] Okonechnikov K, Conesa A, García-Alcalde F (2016). Qualimap 2: advanced multi-sample quality control for high-throughput sequencing data. Bioinformatics.

[46] Seemann T (2014). Prokka: rapid prokaryotic genome annotation. Bioinformatics.

[47] Jolley KA, Maiden MCJ, BIGSdb (2010). Scalable analysis of bacterial genome variation at the population level. BMC Bioinformatics.

[48] Lakin SM, Dean C, Noyes NR, Dettenwanger A, Ross AS, Doster E, et al. MEGARes: an antimicrobial resistance database for high throughput sequencing. Nucleic Acids Res. 2017;45(D1):D574–80. 10.1093/nar/gkw1009. 10.1093/nar/gkw1009 PMC5210519 27899569

