# Correction to: Prevalence and mechanisms of antibiotic resistance in *Escherichia coli* isolated from mastitic dairy cattle in Canada

**DOI:** 10.1186/s12866-021-02301-3

**Published:** 2021-09-06

**Authors:** Satwik Majumder, Dongyun Jung, Jennifer Ronholm, Saji George

**Affiliations:** 1 Department of Food Science and Agricultural Chemistry, McGill University, Macdonald Campus, 21111 Lakeshore Ste Anne de Bellevue, Quebec, H9X 3V9 Canada; 2 Department of Animal Science, McGill University, Macdonald Campus, 21111 Lakeshore Ste Anne de Bellevue, Quebec, H9X 3V9 Canada

**Correction to: BMC Microbiol 21, 222 (2021)**

**https://doi.org/10.1186/s12866-021-02280-5**

Following the publication of the original article [1], we were notified that the following typos have been introduced during production:

In the Results section:

1. ‘isolate 40,816,739 (with the slowest extrusion), and QC strain (with non-functional AcrAB-TolC)’ should read ‘isolate 40816739 (with the slowest extrusion), and QC strain (with non-functional AcrAB-TolC)’.

In the Discussions section:

1. ‘The isolates 10,800,294 and 21,914,232 showed resistance to cefazolin and cefotaxime without ESBL’ should read ‘The isolates 10800294 and 21914232 showed resistance cefazolin and cefotaxime without ESBL’
2. ‘Isolate 10,800,294 was a strong biofilm former and had an active AcrAB-TolC’ should read ‘Isolate 10800294 was a strong biofilm former and had an active AcrAB-TolC’.

In the Materials and Methods section:

1. ‘E. coli ATCC 25,922, S. aureus ATCC 25,923, and P. aeruginosa ATCC 27,853 (Oxoid company, Canada)’ should read ‘E. coli ATCC 25922, S. aureus ATCC 25923, and P. aeruginosa ATCC 27853 (Oxoid company, Canada)’.
2. ‘PPB without cell suspension was used as blank and E. coli ATCC 25,922 was used as a control’ should read ‘PPB without cell suspension was used as blank and E. coli ATCC 25922 was used as a control’.
3. ‘E. coli ATCC 25,922 was used as a control strain to check the difference in biofilm formation’ should read ‘E. coli ATCC 25922 was used as a control strain to check the difference in biofilm formation’.

The original article has been corrected.

## References

[1] Majumder (2021). Prevalence and mechanisms of antibiotic resistance in *Escherichia coli* isolated from mastitic dairy cattle in Canada. BMC Microbiol.

