# Supplementary material for: Prevalence and mechanisms of antibiotic resistance in Escherichia coli isolated from mastitic dairy cattle in Canada
Source: BMC Microbiol. 2021 Jul 31;21:222. doi: 10.1186/s12866-021-02280-5 (PMC8325273; doi:10.1186/s12866-021-02280-5)
Supplement: Supplementary file 1 — Additional file 1. [file 12866_2021_2280_MOESM1_ESM.docx]

**Prevalence and mechanisms of antibiotic resistance in *Escherichia coli* isolated from mastitic dairy cattle in Canada**

Satwik Majumder^a^, Dongyun Jung^a^, Jennifer Ronholm^a,b*^, Saji George^a*^

[satwik.majumder@mail.mcgill.ca](mailto:satwik.majumder@mail.mcgill.ca)

[dongyun.jung@mail.mcgill.ca](mailto:dongyun.jung@mail.mcgill.ca)

[jennifer.ronholm@mcgill.ca](mailto:jennifer.ronholm@mcgill.ca)

^a^McGill University, Department of Food and Agricultural Chemistry, Macdonald Campus, 21111 Lakeshore Ste Anne de Bellevue, Quebec H9X 3V9, Canada

^b^McGill University, Department of Animal Science, Macdonald Campus, 21111 Lakeshore Ste Anne de Bellevue, Quebec H9X 3V9, Canada

*Corresponding authors

Running Title: AMR in *E. coli* isolates from mastitic dairy cattle

Address of correspondence

Department of Food Science and Agricultural Chemistry,

Macdonald-Stewart Building, Room-1039, Macdonald Campus, McGill University

21,111 Lakeshore, Ste Anne de Bellevue, Québec, H9X 3V9, Canada.

Tel: 514-398-7920, Fax: 514-398-7990

Email: [saji.george@mcgill.ca](mailto:saji.george@mcgill.ca)

**
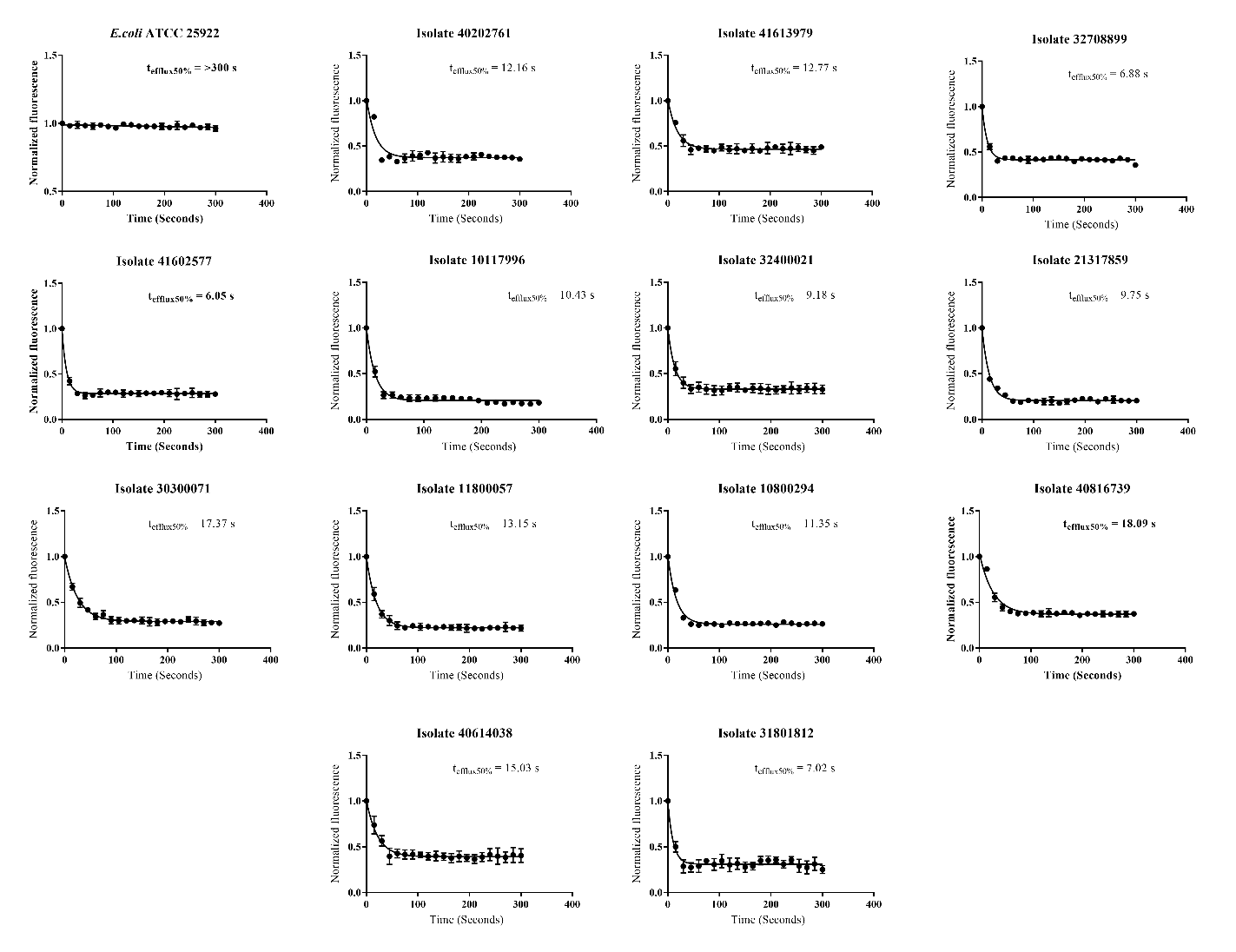
**

**Figure S1.** **Representation of the efflux efficiency implying a functional AcrAB-TolC efflux gene in all 13 *E. coli* isolates after re-energizing cells by adding 25mM of glucose.**


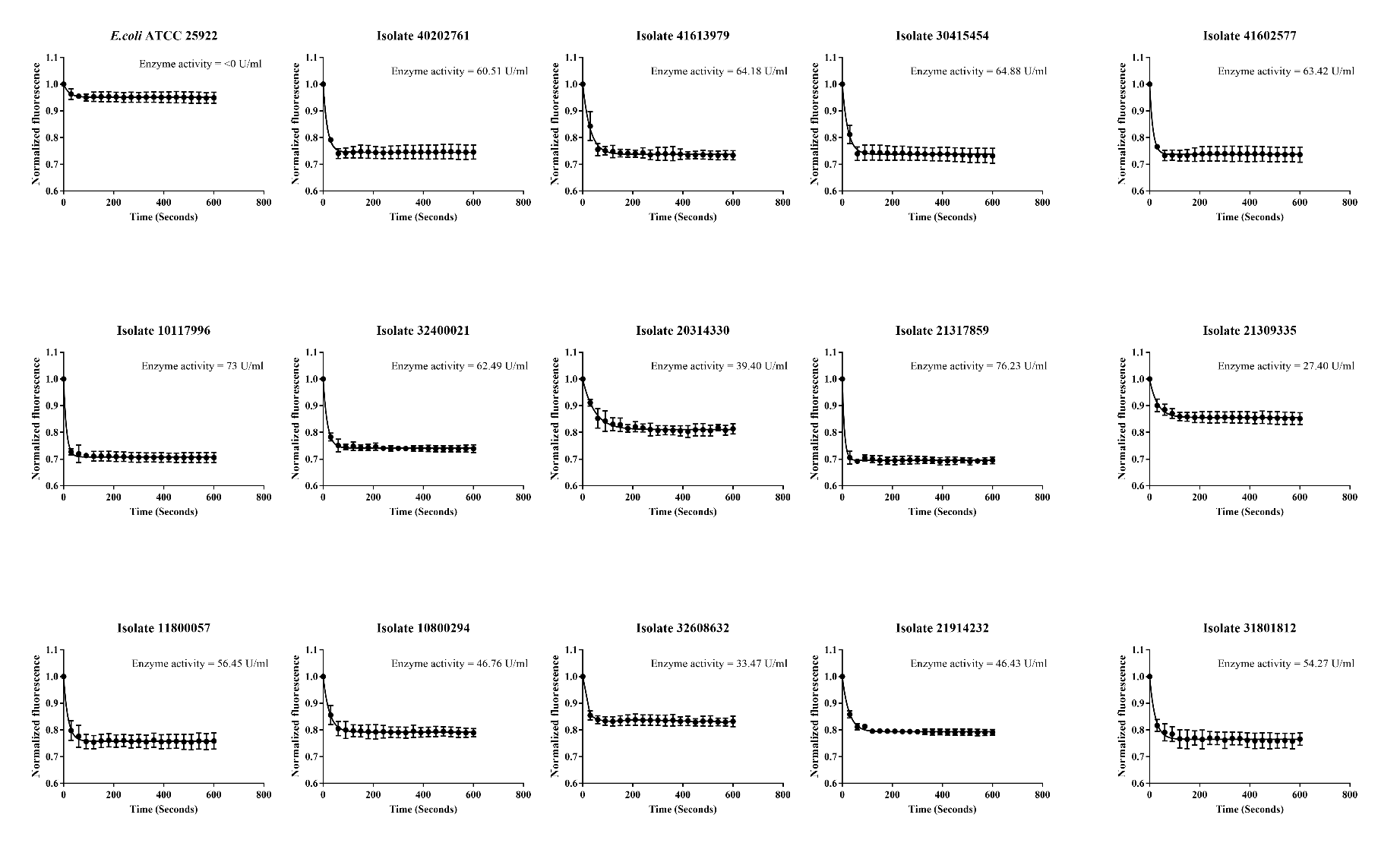


**Figure S2. Depiction of the nitrocefin hydrolysis with respect to time implying the ß-lactamase enzymatic activity induced by 14 *E. coli* isolates.**


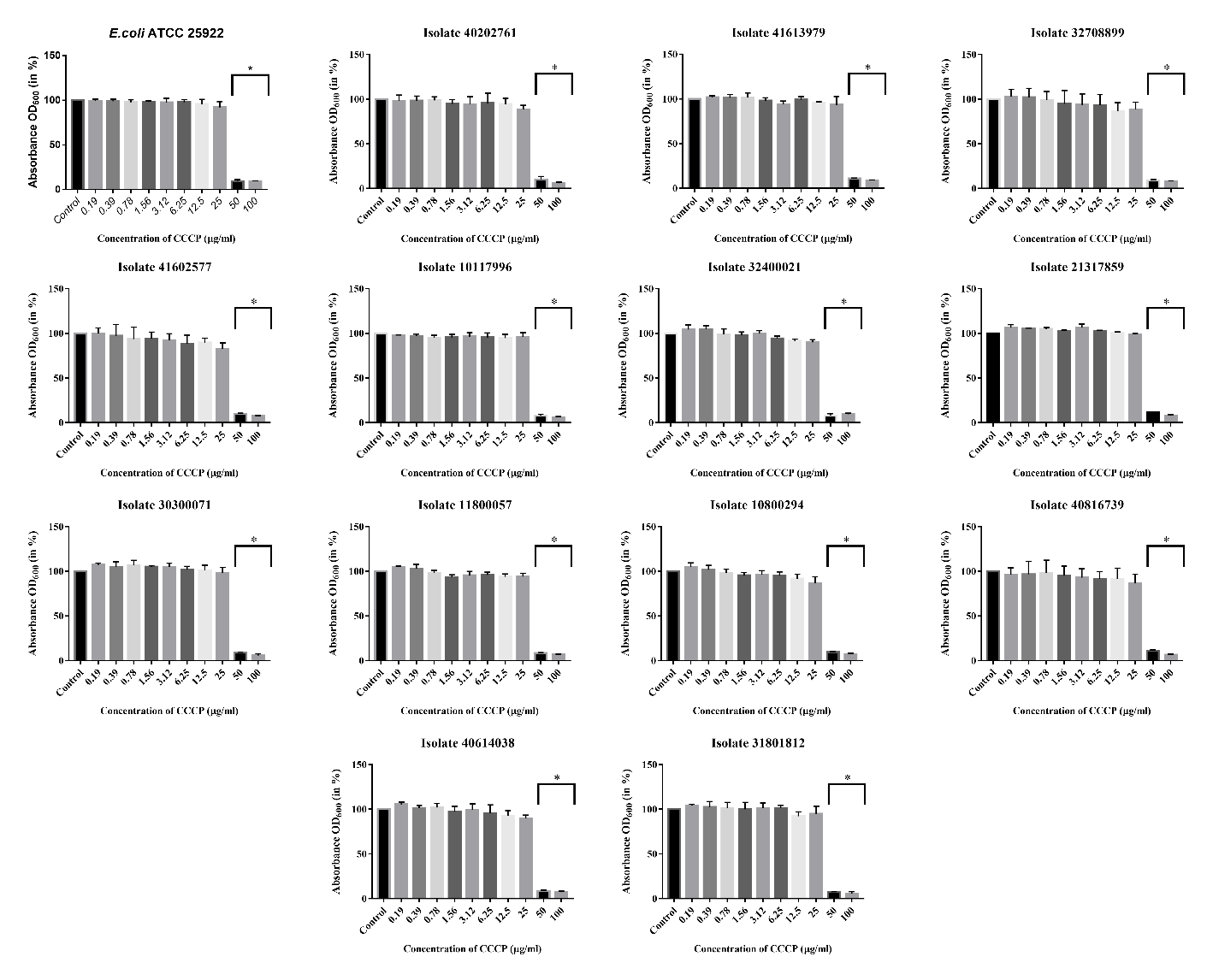


**Figure S3**. **Representation of the MIC (50 µg/ml) of CCCP against 13 *E. coli* isolates and QC strain at OD_600_.** Ten-twofold serial dilutions of the CCCP (from 100 µg/mL to 0.19 µg/mL) were prepared in 100 µL of MH broth in 96 well plates. Ten microliters of pure bacterial cultures maintained at 0.5 McFarland standard were subjected to the wells. The plates were incubated and read at OD_600_ using a plate reader. The study was done to ensure the engagement of CCCP as an anti-biofilm agent rather than interfering in bacterial viability. Asterisks indicate a significant decrease in bacterial viability when compared with the control. One-way ANOVA was performed to check the statistical significance of the obtained data where p-value <0.05 was considered as significant.


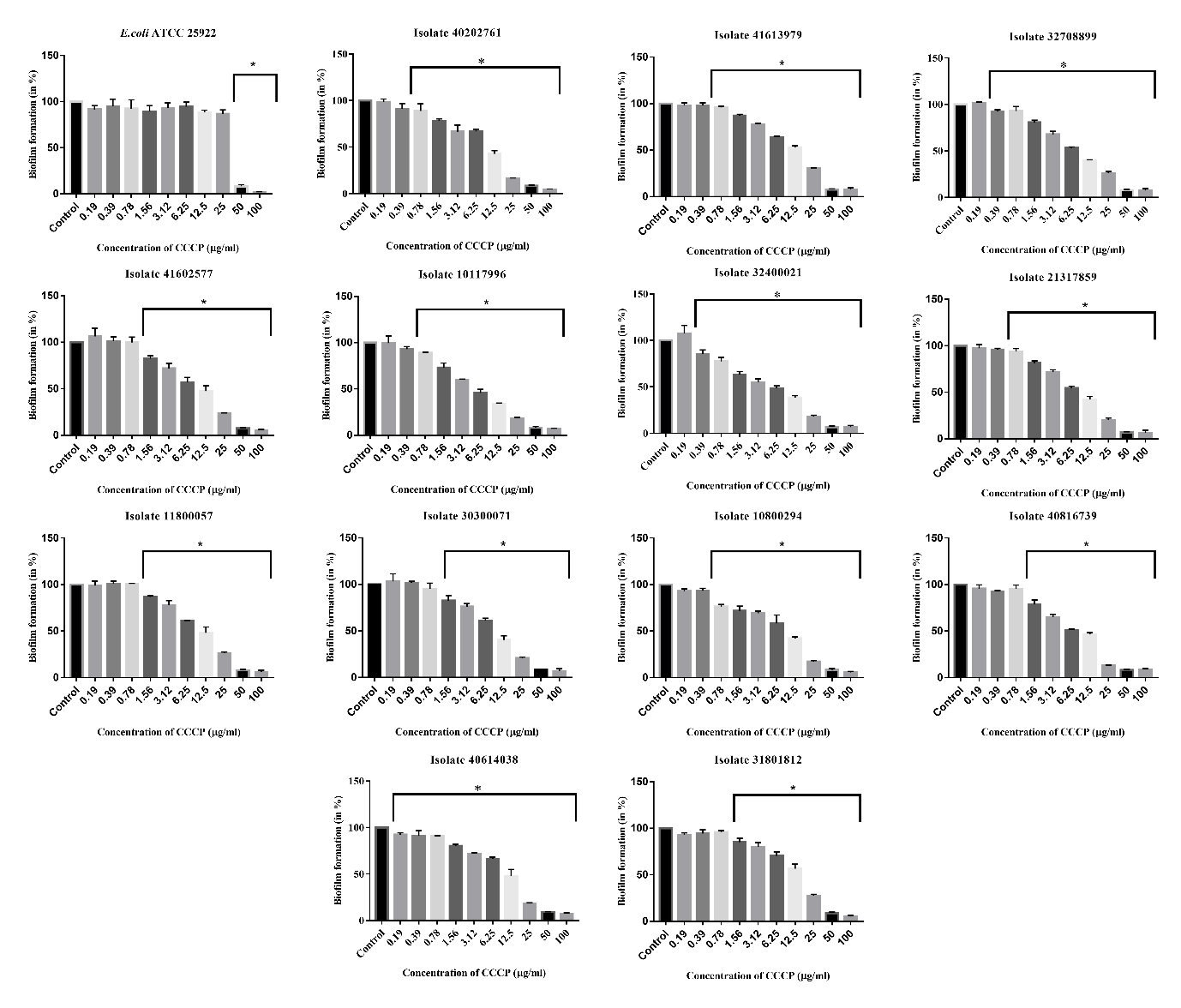


**Figure S4. Representation of the impact of efflux inhibitor, CCCP on the ability of 13 *E. coli* isolates to form biofilms.** One-way ANOVA was performed to check the statistical significance of the obtained data where p-value <0.05 was considered as significant. GraphPad Prism 7 software was used to carry out the statistical analysis and for graphical representation.
